# Resource‐dependent evolution of female resistance responses to sexual conflict

**DOI:** 10.1002/evl3.153

**Published:** 2020-01-09

**Authors:** Wayne G. Rostant, Janet S. Mason, Jean‐Charles de Coriolis, Tracey Chapman

**Affiliations:** ^1^ School of Biological Sciences University of East Anglia Norwich NR4 7TJ United Kingdom

**Keywords:** Diet manipulation, experimental evolution, lifespan, maintenance, resistance to harm, sexual conflict, trade‐off

## Abstract

Sexual conflict can promote the evolution of dramatic reproductive adaptations as well as resistance to its potentially costly effects. Theory predicts that responses to sexual conflict will vary significantly with resource levels—when scant, responses should be constrained by trade‐offs, when abundant, they should not. However, this can be difficult to test because the evolutionary interests of the sexes align upon short‐term exposure to novel environments, swamping any selection due to sexual conflict. What is needed are investigations of populations that are well adapted to both differing levels of sexual conflict and resources. Here, we used this approach in a long‐term experimental evolution study to track the evolution of female resistance to sexual conflict in the fruit fly *Drosophila melanogaster*. In resource‐rich regimes, high‐conflict females evolved resistance to continual exposure to males. There was no difference in baseline survival, consistent with the idea that responses evolving under nutritional abundance experienced no trade‐offs with resistance. In the poor resource regimes, the ability of high‐conflict females to evolve resistance to males was severely compromised and they also showed lower baseline survival than low‐conflict females. This suggested high‐conflict females traded off somatic maintenance against any limited resistance they had evolved in response to sexual conflict. Overall, these findings provide experimental support for the hypothesis that evolutionary responses to sexual conflict are critically dependent upon resource levels.

**Impact summary**

Sexual conflict results from differences in the evolutionary interests of the sexes or sex functions. It is a powerful force for shaping the evolution of reproductive traits, mating systems, and ultimately reproductive isolation, with the potential to create new species. Recent studies highlight a pressing need to incorporate more ecology into the study of sexual conflict, specifically to incorporate the influence of resource levels (e.g., food) and the impact this has on an individual's condition. This is of fundamental importance because resources such as food are expected to significantly influence and even reverse the outcomes of sexual conflict. However, there is a major problem in measuring the effect of resources on the expression of sexual conflict. By placing both sexes in new resource environments, selection is altered: the evolutionary interests of both sexes become aligned and swamped by the need to adapt to the new environment. In this situation, the intensity of sexual conflict becomes contingent not on resources per se, but on the extent to which individuals are adapted to the new environment. Hence a new approach is required to conduct ecological tests of the importance of sexual conflict. Here, we used experimental evolution to simultaneously manipulate sexual conflict and resource levels to allow the first direct tests of this hypothesis. The results showed that responses to sexual conflict evolved in females under nutrient rich but not poor resource levels. This suggests that resource availability can constrain the expression of responses to elevated sexual conflict and in doing so reveal underlying trade‐offs. The work highlights the key, general role of resources such as food in determining the expression of responses to selection.

Sexual conflict results from differences in the evolutionary interests of the sexes or sex functions (Charnov 1979; Parker 1979). It is a powerful force for shaping the evolution of reproductive traits, mating systems and, ultimately, reproductive isolation (Chapman et al. 2003; Arnqvist and Rowe 2005; Bonduriansky and Chenoweth 2009; Mank 2017; Queller and Strassmann 2018). The powerful evolutionary forces involved can be revealed by manipulative experiments to highlight the effect of elevating sexual conflict in one sex upon responses in the other (Rice 1992; Rice 1996; Arnqvist and Rowe 2002; Wigby and Chapman 2004; Ronn et al. 2007). This can reveal the evolutionary to and fro of response and counter response that is characteristic of sexual conflict in action (Chapman 2018). Counter responses may often evolve to minimize potentially costly side effects. For example, an evolutionary history of elevated sexual conflict can lead to the evolution of resistance to costs of mating in females (Wigby and Chapman 2004). In this scenario, females exposed over time to elevated courtship and mating frequencies evolve the ability to ameliorate these effects such that they can express higher survival when continually exposed to males. This suggests the existence of significant standing genetic variation for survival resistance (Long and Rice 2007; Wigby and Chapman 2004), rather than the emergence and spread of new mutations (due to the relatively limited numbers of generations involved).

However, there is a pressing need to better incorporate life history theory and ecology into the study of sexual conflict, and better recognize and emphasize the influence of resource levels (Arbuthnott et al. 2014; Perry et al. 2017; Perry and Rowe 2018) and condition dependence (Rowe and Houle 1996; Chen and Maklakov 2012; Zajitschek and Connallon 2017). Importantly, theory shows that the extent to which sexual conflict is evident will depend upon the genetic variances and between‐sex covariances for resource acquisition and allocation, condition‐dependent expression of resource allocation, and sex differences in selection on the allocation of resource to different fitness components (Zajitschek and Connallon 2017). This is of fundamental importance because resource levels may significantly influence and even reverse the outcomes of sexual conflict (Poissant et al. 2010; Adler and Bonburiansky 2014; Connallon and Clark 2014; Connallon and Hall 2016; Han and Dingemanse 2017). However, to date, we lack empirical tests of this key idea. In addition, differences in the manifestation of conflict could also be influenced by sex differences in resource acquisition. For example, high levels of dietary protein can increase costs associated with mating in females (Chapman and Partridge 1996; Lee et al. 2008; Schultzhaus and Carney 2017), which may be at least partly attributable to higher female feeding rates (Lee et al. 2013). The requirement to broaden our understanding of sexual conflict also reflects the wider recognition of the importance of resource levels in directing the magnitude and sign of trade‐offs within an organism's life history (e.g., Calow 1982; Reznick 1985; van Noordwijk and de Jong 1986; Roff 1992; Stearns 1992; Reznick et al. 2000; Roff and Fairbairn 2007; Gray et al. 2018). Direct manipulations of diets are generally seen as a powerful way in which to investigate the effect of resource levels on trade‐off expression (Reznick 1985; Reznick et al. 2000; Roff and Fairbairn 2007; Rapkin et al. 2018), an approach that has been used across a variety of different taxa (e.g., invertebrates, Hunt et al. 2004; fish, Kolluru and Grether 2004; Grether et al. 2005; amphibians, Lardner and Loman 2003; Stuart et al. 2019; reptiles, Telemeco and Baird 2011; birds, Karell et al. 2008; and mammals Hill and Kaplan 1999; Forbes et al. 2014).

A particular challenge in measuring the effect of resource levels on the expression of sexual conflict is that by placing both sexes in new resource environments, the nature of selection changes such that the evolutionary interests of the sexes can become aligned, or effectively swamped, by direct selection upon both sexes to adapt to the new environment. Consistent with this, comparisons of “poorly” adapted populations, that is, those subjected to novel temperature stress, diets/diet choices, generally show less sexual antagonism than under the standard conditions to which they are “well adapted” (Maklakov et al. 2009; Fricke et al. 2010; Long et al. 2012; Reddiex et al. 2013; Berger et al. 2014; Han and Dingemanse 2017). Similarly, Arbuthnott et al. (2014) show that the exaggeration of traits under sexual conflict tended to be limited in environments in which natural selection on these traits was stronger, whereas environments with weaker natural selection allowed for greater trait exaggeration (see also Rowe et al. 2003). Hence, the intensity of sexual conflict is expected to be contingent not on resource levels *per se*, but upon the extent to which individuals are adapted to their environment (Connallon and Clark 2014). Direct tests of the significance of resources require comparisons of the extent of sexual conflict in populations that are already well adapted to different conditions.

Here, we applied experimental evolution to conduct a novel test of the effect of resource levels on evolutionary responses to sexual conflict. We used experimental evolution to produce lines in which sexual conflict was elevated or decreased under resource‐rich or resource‐poor nutritional conditions (Supporting Information). We focused on the evolutionary resistance responses of females (Wigby and Chapman 2004) and created regimes in which females had an evolutionary history of exposure to high or low levels of sexual conflict (i.e., male‐biased [MB] or female‐biased [FB] regimes in which courtships and matings per female were elevated or decreased, respectively). Simultaneously, we imposed two resource level treatments for the adults of all regimes: resource‐rich (100 g/L yeast) or resource‐poor (20 g/L yeast) adult diets (Supporting Information). The developmental diet during experimental evolution was standardized (on the 100 g/L yeast) across all diet regimes and conflict levels.

This allowed us to test, in well‐adapted populations experiencing similar selection pressures, the hypothesis that resource levels constrain female responses to elevated sexual conflict. High‐conflict females from rich‐resource regimes, unconstrained by trade‐offs with somatic maintenance, are expected to have the capacity to express resistance responses. In contrast, under poor resource regimes, such females are expected to evolve resistance to sexual conflict only by trading it off against somatic maintenance. Hence, our first prediction was that elevated sexual conflict would restrict the evolution of male harm resistance to resource‐rich regimes, because only in these would females have the capacity to respond to selection by investing in somatic maintenance and resistance. The general lack of male survival responses to sexual conflict observed in our previous study (e.g., Wigby and Chapman 2004) and the finding that life history traits in males can show limited responses to proximate diets (e.g., Magwere et al. 2004) generated a second prediction: that the outcomes would be sex specific. To test these predictions, and determine the full spectrum of female fitness responses, we conducted standardized assays to determine lifespan, ageing, and fitness for both sexes of each regime on the same diet, under “no conflict” (once‐mated individuals—to reveal investment in base line somatic maintenance) and “conflict” (continually mated individuals—to indicate investment in resistance) assay conditions.

## Results and Discussion

Our experimental rationale for testing the hypothesis that resource levels had constrained female responses to elevated sexual conflict was to (1) infer the nature of the selective environment experienced by measuring the key drivers of courtship and mating frequency within all treatments during the experimental evolution, and (2) deploy standardized lifespan and fitness assays for all regimes, on the same diet and reproductive conditions, in “conflict” and “no conflict” assays. This allowed direct comparisons of lifespan, ageing, and fitness in individuals derived from the rich and poor, high, medium, and low sexual conflict regimes. Replicated measurements taken at different generations during selection also allowed us to track the trajectory of evolutionary responses over time.

### SELECTIVE ENVIRONMENT EXPERIENCED IN THE RICH AND POOR RESOURCE SEXUAL CONFLICT REGIMES

Measurements of the selective environment during experimental evolution showed that, as expected, females from the high‐conflict (i.e., MB) regimes experienced significantly elevated courtship and mating in comparison to the medium conflict equal sex (ES) and low‐conflict (FB) females, and that this was maintained over time (Supporting Information; Fig. S1A,B). This suggested that the intensity of selection arising from sexual conflict was similar for any given sex ratio on either resource regime. However, it is important to note that we did not have direct measures of the strength of selection in females. That said, courtship and mating frequency behaviors measured as our indirect proxy for selection appear to be key drivers of female resistance (Wigby and Chapman 2004). Hence, as these indices of conflict were similar within sex ratio treatments across resource levels, it is reasonable to infer that key selective forces driving female resistance were also similar. We cannot rule out other possibilities that have not yet been tested, for example, that a high condition male from the rich diet regime might harm a female more at each mating than a corresponding poor diet regime from low condition male. Direct tests of this idea have not yet been conducted, although investigations of the proximate nutritional effects of a male's ability to effect postmating responses in females (Fricke et al. 2008) showed no evidence that male nutrition at the levels imposed here affected the relative share of paternity of first or second mating males, though there were some effects on the ability of the first mating males to induce remating refractoriness in females. Resource‐poor flies spent less time on the food (Fig. S1C) and within the rich food regimes, high‐conflict (MB) flies showed lower food occupancy. This suggests that the high frequency of male courtship and mating could have prevented females from accessing the food, potentially compounding any harmful effects of males on females.

### RESPONSE OF LIFESPAN, AGEING, AND MATING FREQUENCY TO SEXUAL CONFLICT IN RICH AND POOR RESOURCE REGIMES

**Figure 1:**
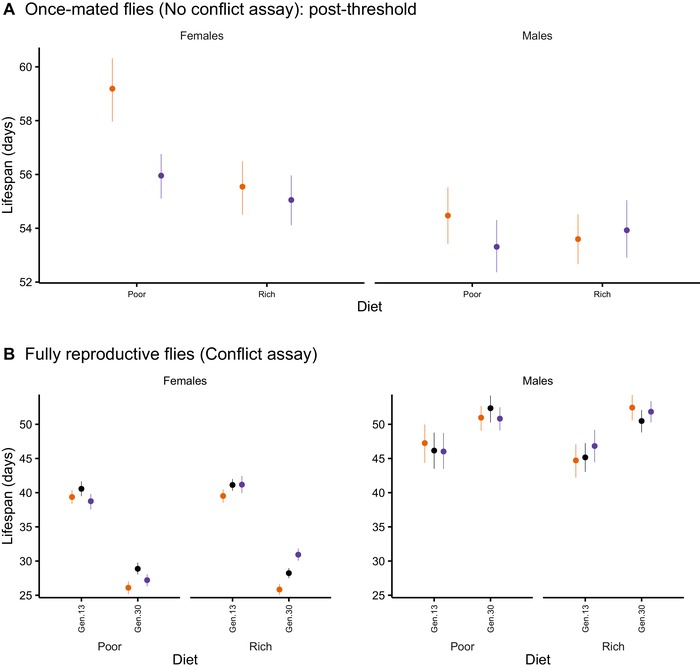
Response of survival to manipulation of sexual conflict and resource levels under no conflict (A) and conflict (B) assay conditions. Mean (±95% bootstrap CI) lifespan for males and females is shown. (A) For flies which survive past age 32 days, average lifespan of male‐biased (MB, purple) and female‐biased (FB, orange) treatment once‐mated females and males from resource‐poor and resource‐rich regimes following experimental evolution (generation 47) (no conflict assay conditions). This panel shows the higher base line survival in the low‐conflict (FB) in comparison to high‐conflict (MB) females from the poor resource diet regime, whereas in females from the rich diet regime there was no difference. Male survival showed minimal evolutionary responses to diet regime and sex ratio. (B) Mean lifespan of MB (purple), equal sex (ES, black), and FB (orange) treatment fully reproductive females and males derived from the poor and rich diet regimes at generation 13 and 30 of experimental evolution (conflict assay conditions). This panel highlights the increase in sex differences in lifespan as experimental evolution proceeded, and the response of female lifespan in rich, but not poor, diet regimes to sexual conflict, with MB > ES > FB. Male lifespan did not respond to either evolutionary diet regime or sex ratio.

1. *Somatic maintenance—baseline survival and ageing in “no conflict assay” conditions*: We first analyzed the survival of once‐mated individuals at generation (gen) 47 under “no conflict” assay conditions. Individuals were mass mated at the start of the experiments (placed with wild‐type individuals of the opposite sex for 24 hours) and then all maintained on standard SY (rich) medium in single‐sex vials until death. Low‐conflict (FB) females lived longer than high‐conflict females (MB) in the poor but not rich resource regimes (Fig. S2A), consistent with the prediction that females with an evolutionary history of high conflict in poor regimes had compromised somatic maintenance. There appeared to be no effects of sex ratio on male lifespan. However, the distribution of lifespan (Fig. S2A) was strongly left skewed, which motivated a two‐step model (Supporting Information) consisting of survival to a threshold (@32 days, Fig. S3) followed by lifespan analysis of the majority of the cohorts that formed the postthreshold survivors (Fig. 1A).

*Threshold survival*: Most individuals survived the threshold period, hence the major longevity signal came from the postthreshold analysis described below. Nevertheless, within the threshold period, high‐conflict (MB) females (survival probability = 0.919 [0.895, 939]) had significantly lower threshold survival (*Z* = 3.107, *P*_adjust_ = 0.0076) than did MB males (survival probability = 0.963 [95% CI = 0.944, 0.975]), whereas there was no sex difference in the FB regimes (female survival probability = 0.944 [0.922, 0.960]; males = 0.929 [0.905, 0.947]; *Z* = 1.064, *P*_adjust_ = 0.2874). A significant interaction between sex ratio and focal sex (LRT = 9.3977, *P* = 0.0027) was also evident, due to higher survival in high‐ (MB) over low‐conflict (FB) regime males (*Z* = 2.535, *P*_adjust_ = 0.0224). There was no effect of evolutionary resource regime on threshold survival across any sex ratio treatment (LRT = 0.6016, *P* = 0.4403). The results suggested that high, but not low, conflict females had reduced threshold survival relative to their respective males, consistent with the prediction that their somatic maintenance was compromised (Fig. S3).

*Postthreshold survival*: Approximately 94% of all uncensored individuals survived to day 32 and thus formed the postthreshold cohorts (Fig. 1A). We observed significant interactions of focal sex with sex ratio (LRT = 4.5079, *P* = 0.0349) and focal sex with diet regime (LRT = 9.6881, *P* = 0.0013). Low‐conflict (FB) females were longer lived than high‐conflict (MB) females, but this was marginally significant only in individuals derived from the poor resource regime (*P*_adjust_ = 0.0516). Overall, low‐conflict (FB) females had consistently, but not significantly, longer postthreshold lifespans than MB females across both resource regimes (FB–MB lifespan ± SEM = 1.91 ± 1.42 days; *t*_10.2_ = 1.344, *P*_adjust_ = 0.2929). Focal sex was significant (LRT = 53.024, *P* < 0.0001) with females having significantly longer lifespans than males, especially in individuals from the low‐conflict (FB) poor resource regimes (female–male lifespan ± SEM = 4.37 ± 0.60 days; *t*_2178.27_ = 7.274, *P*_adjust_ < 0.0001; Fig. 1A).

We suggest that FB females from the poor regimes lived longer than the other females in the no conflict assay because their selective environment was dominated by resource levels experienced during the experimental evolution. Therefore, the response of FB female lifespan was strongly determined by the poor food levels they experienced, and, of the four female treatments these females were most likely to evolve mechanisms to support somatic maintenance. This was the converse of the MB females maintained on rich food regimes whose selective environment was dominated not by paucity of food but by male attention/harm. These females responded to the force that most affected fitness by resisting harm (see below). This was supported by the mirrored responses in the FB and MB females (comparing no conflict assay at 47 generations and conflict assay at 30 generations) shown in Fig. 1A versus B. Just as FB females from poor food regimes were outliers in the “no conflict assay,” MB females from the rich food regimes were outliers compared to all other females in the “conflict assay.”

**Figure 2:**
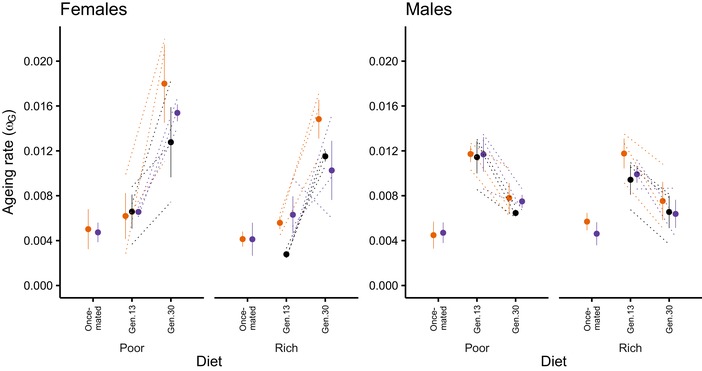
Response of ageing and fitness to manipulation of sexual conflict and resource levels under no conflict and conflict assay conditions. Rate of ageing (mean of replicates ± 95% CI) in once‐mated (no conflict assay) females and males from the FB (orange) and MB (purple) treatments (generation 47) from the poor and rich diet regimes and for fully reproductive (conflict assay) females and males from the MB, ES (black), and FB treatments on both diet regimes at generation 13 and 30 of experimental evolution. The lack of response of ageing in the once‐mated low (FB) and high (MB) conflict individuals is evident. The opposing pattern of ageing rate evolution as experimental evolution proceeded is evident in the fully reproductive individuals, as is the response of ageing to sex ratio regime in females from the rich (MB > ES > FB), but not poor diet regime and the lack of responses in males.

*Ageing rate*: None of the treatments significantly affected the ageing profile (Figs. S4A,B, S5A) or ageing rate (*ω_G_*) (Fig. 2). At first sight, this might appear to contradict the significant effects observed in the analysis of postthreshold lifespan. However, the ageing rate analysis was done at the population scale, with ageing parameters derived from summaries of ∼100 individuals’ longevity data. This may have decreased power and the resolution to detect differences, particularly given the variability between populations in survival curve shapes, which might mask different population‐level trajectories within the same treatments.

Overall, the results were consistent with the prediction that, under the poor resource regimes, investment in somatic maintenance (lifespan under no conflict conditions) of low‐conflict (FB) females was relatively unconstrained (no trade off with resistance), whereas that of high‐conflict females from the MB experimental evolution regimes was compromised. Why the rich regime females did not live longer overall is not fully clear, although we suspect there was stronger viability selection related to resource use efficiency in the poor resource regime.

2. *Evolution of resistance—Survival, ageing, and mating in fully reproductive flies (“conflict assay” conditions)*. We then analyzed survival patterns in both sexes under fully reproductive, “conflict assay” conditions (i.e., with focal individuals exposed continually in a 50:50 sex ratio to standard wild‐type individuals of the opposite sex, and all maintained on the standard [rich] diet). Two generational timepoints, 13 and 30, were measured.

*Lifespan*: Although we found no significant three‐way interactions, survival was significantly impacted by two‐way interactions involving generation, sex ratio treatment, and sex (sex ratio × diet, LRT = 9.8897, *P* = 0.0467; sex ratio × focal sex, LRT = 11.9907, *P* = 0.0042; focal sex × generation, LRT = 436.63, *P* = 0.0012). There were also highly significant main effects of generation (LRT = 67.229, *P* < 0.0001) and focal sex (LRT = 1608.6, *P* < 0.0001). To further probe the interactions, we split the dataset by diet regime and subsequently re‐analyzed each subset separately.

In individuals derived from the resource‐rich regimes, lifespan changed significantly over time (generation × focal sex, LRT = 277.04, *P* = 0.0010) and was differentially impacted by sex ratio treatment (sex ratio × focal sex, LRT = 13.2755, *P* = 0.0040). Here, high‐conflict MB females had significantly longer lifespans than either medium‐ (ES) or low‐conflict (FB) females (FB < ES: *t*_11.02_ = –3.823, *P*_adjust_ = 0.0056; FB < MB: *t*_11.24_ = –6.477, *P*_adjust_ = 0.0001; ES < MB: *t*_10.97_ = –2.684, *P*_adjust_ = 0.0341; Fig. 1B and Figs. S2B, S4C,D). In contrast, in the poor diet regimes, although lifespan was explained by an interaction between focal sex and generation (LRT = 172.68, *P* = 0.0010) and by main effects of focal sex (LRT = 857.21, *P* = 0.0010) and generation (LRT = 33.139, *P* = 0.0010), we found no effect of sex ratio on female lifespan. The results showed that only high‐conflict (MB) females from the rich resource regimes could apparently evolve resistance, that is, mechanisms that could ameliorate the deleterious impacts of elevated sexual conflict. The concordance of this lifespan response with a previous, independently conducted experimental evolution experiment (Wigby and Chapman 2004) demonstrates the robustness of this result. In neither evolutionary diet regime did we find an effect of sex ratio on male lifespan (FB = ES = MB, all *P*_adjust_ ≫ 0.05).

Across both resource levels regimes, we also saw a striking increase in sexual dimorphism in lifespan as the experimental evolution proceeded (Fig. 1B, Fig. S2B). This appeared to be a side effect of the husbandry regime (Supporting Information). Exactly why is not yet clear, but it could reflect sex‐specific trade‐offs (Fairbairn 2013) in response to selection to reproduce maximally at day 10. This effect was more pronounced in the rich diet regime, where the significant main effect of focal sex (LRT = 19.1988, *P* = 0.0022) was due to females having significantly shorter lifespans than males. The significant focal sex × generation effect was driven by a 6‐day increase in male (gen 13 mean ± SEM = 47.99 ± 0.64; gen 30 = 53.70 ± 0.52; *t*_193.07_ = –6.806, *P* < 0.0001) and simultaneous 12‐day decrease in female lifespan (gen 13 mean ± SE = 41.52 ± 0.27; gen 30 = 29.21 ± 0.53; *t*_15.07_ = 20.110, *P* < 0.0001).

*Ageing parameters* (α, β): Ageing parameters showed significant main effects of focal sex (*F*_1,66_ = 50.646, *P* < 0.0001), diet (*F*_1,66_ = 4.672, *P* = 0.0027), generation (*F*_1,66_ = 0.961, *P* < 0.0242), and sex ratio (*F*_2,66_ = 2.084, *P* < 0.0304). During model simplification, the focal sex × generation interaction became significant (*F*_1,58_ = 37.175, *P* < 0.0001) attributable to a decrease in the initial mortality rate (α) of males between generation 13 and 30, whereas the opposite occurred in females (Fig. S5B). Thus, females had a consistently higher rate of mortality increase. There was a marginally higher initial mortality rate in the poor diet regime populations and a slight increase in the rate of mortality across generations (Fig. S5B). Overall, low‐conflict (FB) regimes had higher initial mortality and lower mortality rate increase than did ES or high‐conflict (MB) regimes. Analysis of the ageing rate parameter (ω_G_) revealed a main effect of diet regime (LRT = 4.322, *P* = 0.04794) with poor resource regime flies ageing faster than those from rich regimes, and a single significant interaction (focal sex × generation, LRT = 66.034, *P* = 0.0001). Female ageing was initially lower than for males and increased over time, resulting in a significant decrease in mean lifespan. Meanwhile, male ageing decreased over time (Figs. S2B, S5B). When this interaction was removed, there was a significant main effect of generation (LRT = 4.729, *P* = 0.0317). By generation 30, ageing in females correlated inversely with the level of sexual conflict (with the ageing rate parameter ω_G_ higher in FB > ES > MB; Fig. 2A), although this effect was nonsignificant due to the reduced power of the population‐level ageing analysis. The pattern was consistent with the survival analysis and supported the existence of resource‐dependent resistance to male‐induced harm.

**Figure 3:**
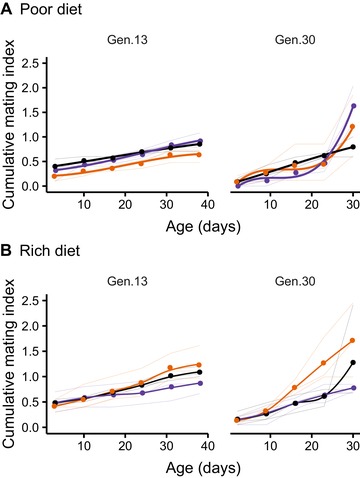
Cumulative, age‐specific female mating rate over the lifetime. Shown are females from the MB (purple), ES (black), and FB (orange) (A) poor and (B) rich diet regimes at generation 13 and 30 of the experimental evolution (conflict assay conditions). The bold colors show the replicate averages, with individual replicate responses shown in the lighter background. The figures show how mating rate increased over the lifetime more markedly in all regimes as experimental evolution proceeded. The increase was significantly elevated in low‐conflict (FB) females (which had lowest survival) derived from the resource‐rich diet regime.

*Mating frequency*: There was no sex ratio × age interaction (LRT = 5.1935, *P* = 0.0932) for mating frequency on resource‐poor regimes at gen 13, but by generation 30 a strong nonlinear effect was evident (LRT = 9.6866, *P* = 0.0055; Fig. 3). High‐conflict (MB) and low‐conflict (FB) regimes showed a significant increase with age, and in MB regimes in particular, a more rapid acceleration (sex ratio × age interaction, LRT = 16.2653, *P* = 0.0011). The corresponding resource‐rich regimes showed a constant mating rate. However, FB rose significantly more steeply than for either ES or MB regimes, resulting in significantly higher mating overall (sex ratio × age interaction, LRT = 7.5074, *P* = 0.0438; Fig. 3), which may be associated with higher mating costs and the observed decreased survival of FB females when exposed to wild‐type males. There was no evidence of any sex ratio × age interactions for courtship.

Collectively, the results of the conflict assays suggested that elevated sexual conflict led to the evolution of resistance in females derived from the resource‐rich regime (Berg and Maklakov 2012; Wigby and Chapman 2004) potentially due to constraints with somatic maintenance (Chapman 2006; Houslay et al. 2015). This was evident as a reduction in the lifespan, and increase in ageing, of fully reproductive low‐conflict (FB) females over ES and high‐conflict (MB) females derived from the resource‐rich regimes, with no such effect in the poor diet regimes. The finding that MB females evolved resistance to the extent that their lifespans actually exceeded that of FB females that experienced low conflict (hence minimal selection for resistance) seems to represent an “overcompensation” in that they survived better than females experiencing low harm. This may suggest that factors in addition to resistance evolution are also involved. Male lifespan was minimally affected by sexual conflict manipulations. The pattern of survival was counter to that seen in the no conflict assay conditions and was underpinned by changes to female ageing under resource‐rich regimes, which showed an inverse correlation with the level of sexual conflict. Hence, for females from the resource‐rich regime, both lifespan and ageing evolved in a manner consistent with the evolution of resistance to male‐induced harm (Wigby and Chapman 2004). Female lifespan upon continual male exposure was also considerably shorter than for once‐mated females, showing strong effects of mating status on female survival (Reiwich and Nuzhdin 2002; Magwere et al. 2004; Liker and Szekely 2005; Maklakov et al. 2009; Barrett and Richardson 2011; Wilson et al. 2013). Although the results suggest that indeed female mating resistance may have been more evolvable under high resources, this is not a formal expectation generated from theory. To support this interpretation, and discount the possibility that at least some portion of responses is due to plastic responses to the increased food levels, it would be very useful to demonstrate greater additive genetic variance in resource acquisition in the high resource treatment as well as estimation of the treatment effects of the relevant evolutionary parameters (e.g., genetic variances).

### RESPONSE OF FITNESS TO VARIATION IN SEXUAL CONFLICT UNDER RESOURCE‐RICH AND RESOURCE‐POOR ENVIRONMENTS

We also analyzed age‐specific reproduction and fitness at gen 13 and 30 for individuals held under the “conflict assay” conditions described above. The gen 13 data showed that, in the poor resource regimes, there was a significant interaction of sex ratio and age (LRT = 18.125, *P* = 0.0001) with a steeper curve for ES in comparison to high‐conflict (MB) and low‐conflict (FB) females (Fig. S6A) and higher initial egg count at 5 days of age (significant interaction of sex ratio × time, LRT = 11.620, *P* = 0.0030). In contrast, under the resource‐rich regime, the ES and FB curves were similar and steeper than for MB females (Fig. S6A) with higher initial fecundity at 5 days of age. An analysis of the day 5 data yielded a significant interaction (LRT = 9.5567, *P* = 0.0084; Fig. S6, insets). Analyses of fitness, calculated as the intrinsic rate of increase in “*r”*, matched this pattern (Fig. S7A). Age‐specific reproduction (offspring) after 30 generations showed no significant effects of sex ratio (Fig. S6B). As above, treatment differences were manifested early, prompting a separate analysis of the day 8–10 data, which represented the 2 days leading up to the window during which fitness is realized during the experimental evolution (day 10–12). However, there were no differences in offspring in the day 10–12 data, which suggests that any “deficit” in offspring in MB females on days 8–10 was not observed during the day 10–12 selection window itself. Our interpretation is that MB females delay peak reproductive effort (steep gradient from day 8–10 rising to day 10–12 offspring) and that it is this ability to more finely tune reproductive schedule to match the selection regime that is adaptive. There was no apparent effect of sex ratio treatment in poor resource regime females or males (Fig. S7B). The early fitness differences observed in females from poor resource lines also disappeared by gen 30, indicating that costs of exposure to high or low frequency of mating or courtship may have been ameliorated by selection.

Overall, the fitness analyses showed that the longer lifespan in fully reproductive high‐conflict (MB) females subjected to elevated sexual conflict was associated with lower early life fitness, indicating a trade off of evolving resistance to males (Arnqvist and Rowe 2005). No such effect was seen in females from the poor resource regime or in males from either nutritional regime. This highlighted that fitness also evolved in a resource‐dependent and sex‐specific manner in response to alteration of sexual conflict (Chapman 2006; Archer et al. 2012; Berg and Maklakov 2012; Archer et al. 2015). The evolutionary diet manipulations were both quantitative (to calorie level) and qualitative (protein to carbohydrate ratio). The rationale was to strongly drive selective responses to overall resource “level.” Whether this type of dietary manipulation itself predisposed to sex‐specific responses is unclear and future evolutionary diet manipulations could target this possibility using more precisely defined diets.

## Conclusions

**Figure 4:**
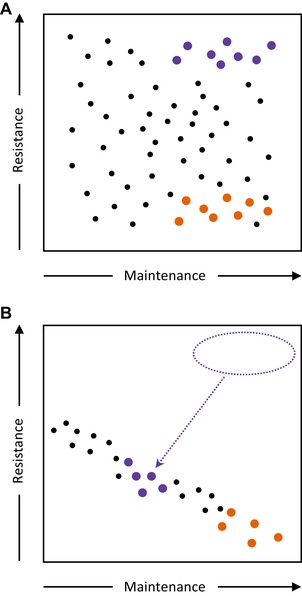
Resource regimes significantly affect phenotype space and the presence or absence of trade‐offs between resistance to sexual conflict and baseline somatic maintenance. (A) For individuals from the rich‐resource diet regime, the potential phenotypic space is wide because unlimited resources mean there is no correlation between expression of resistance and maintenance (indicated by the wide scatter of all dots, each dot indicating a phenotype). Thus, high‐conflict (MB) females can express higher resistance to sexual conflict (longer lifespan when continually exposed to males) than is true for low‐conflict (FB) females. No difference is observed in indices of somatic maintenance (lifespan in the absence of males) under no conflict conditions. (B) For individuals from the poor resource regime, the potential phenotypic space is constrained such that there is now a trade‐off between resistance and maintenance. High‐conflict (MB) females are constrained by this trade off (the shift indicated by the dotted arrow)—they can now express only limited resistance to sexual conflict (shown on Y axis), resulting in minimal difference in lifespan in comparison to low‐conflict (FB) females, when continually exposed to males. Because high‐conflict (MB) females now invest less in somatic maintenance as well, their lifespan in the absence of males is significantly reduced under no conflict conditions (shown on X axis), in comparison to low‐conflict (FB) females.

This study revealed, in line with our prediction, that only females evolving under resource‐rich regimes were able to express responses to minimize the lifespan‐shortening effects of continual exposure to males (Fig. 4). This supported the hypothesis that responses to sexual conflict are resource dependent. The lack of expression of female resistance in individuals derived from the poor resource regimes helped to reveal the underlying trade‐offs with somatic maintenance. Under rich diet regimes, males do not appear to evolve to become more or less harmful to females (Wigby and Chapman 2004; and Fig. S1A,B). Hence, there was no evidence from this current study to suggest that evolution under poor resource levels rendered males less harmful to females. The in situ mating rate remained high over time in the high‐conflict treatments, which instead suggested that females from these regimes had not simply avoided mating frequently, but instead had evolved lower sensitivity to the effects of potentially harmful seminal fluid proteins (Fricke et al. 2013).

Late life survival did not contribute to fitness during the experimental evolution (selection was focused instead on survival to reproduce at day 10–11, Supporting Information). This removed late life costs, which may have enhanced resource investment into manipulative/defensive traits, particularly for the resource‐rich conditions. However, it is also possible that there was some expression of sublethal effects on condition and reproductive ability that contributed to lifespan differences, with higher sexual conflict resulting in higher mortality rates and faster underlying ageing (as observed in the base line survival and ageing of the high‐conflict females from poor resource regimes (Promislow 2003)). The shape of the fitness payoffs between different resource levels, investment in lifespan, and sexual competition can also be complex (Hooper et al. 2017). There may be demands on energetic resources acting as additional constraints (Roff and Fairbairn 2007), for example, associations with immunity (Fabian et al. 2018), which could contribute to the weaker counter adaptation to male harm in the poor resource level regimes. There were no differential responses to sexual conflict observed in male survival or fitness, which was consistent with our second prediction that responses to sexual conflict would follow a sex‐specific pattern. This is consistent with the view that many life history traits in males are significantly less responsive to resource levels than is true for females (e.g., Magwere et al. 2004; Regan and Partridge 2013).

Collectively, the results suggest that the capacity to respond to selection arising from the expression of sexual conflict (Fricke et al. 2008) was condition dependent (Zajitschek and Connallon 2017). Hence individuals subjected to variation in sexual conflict responded to that selection only when resources were nonlimiting, allowing the appropriate phenotypes to be expressed. Recent advances in theory also highlight the importance for sexual conflict of genetic variances and between‐sex covariances for resource acquisition and allocation, and sex differences in allocation of resources to different fitness components (Zajitschek and Connallon 2017). The existence of significant sex‐specific life history responses to sexual conflict that we found here prompt further measurements of the underpinning sex‐specific genetic variation. The current study also adds to a growing body of work showing that aspects of the ecological environment mediate the expression and/or outcome of conflict.

Associate Editor: J. Mank

## Supporting information


**Fig S1**. Frequency of courtship, mating, and food occupancy during experimental evolution under male biased (MB, purple), equal sex (ES, black), and female biased (FB, orange) conditions on poor or rich resource diet regimes.
**Fig S2**. Response of survival to manipulation of sexual conflict and resource levels.
**Fig S3**. Baseline threshold survival to 32 days during no conflict assay.
**Fig S4**. Individual survival curves for females (red curves) and males (blue curves).
**Fig S5**. Ageing parameters, log initial mortality rate (α), and the rate of mortality increase (β) for MB (purple symbols), ES (black), and FB (orange) males and females.
**Fig S6**. Age‐specific fitness for fully reproductive individuals from the MB (purple), ES (Black), and FB (orange) poor and rich diet experimental evolution regimes (conflict assays).
**Fig S7**. Response of fitness to manipulation of sexual conflict and resource levels under conflict assay conditions.
