# Supplementary material for: Resource‐dependent evolution of female resistance responses to sexual conflict
Source: Evol Lett. 2020 Jan 9;4(1):54–64. doi: 10.1002/evl3.153 (PMC7006461; doi:10.1002/evl3.153)
Supplement: Supplementary file 1 — Fig S1. Frequency of courtship, mating, and food occupancy during experimental evolution under male biased (MB, purple), equal sex (ES, black), and female biased (FB, orange) conditions on poor or rich resource diet regimes. Fig S2. Response of survival to manipulation of sexual conflict and resource levels. Fig S3. Baseline threshold survival to 32 days during no conflict assay. Fig S4. Individual survival curves for females (red curves) and males (blue curves). Fig S5. Ageing parameters, log initial mortality rate (α), and the rate of mortality increase (β) for MB (purple symbols), ES (black), and FB (orange) males and females. Fig S6. Age‐specific fitness for fully reproductive individuals from the MB (purple), ES (Black), and FB (orange) poor and rich diet experimental evolution regimes (conflict assays). Fig S7. Response of fitness to manipulation of sexual conflict and resource levels under conflict assay conditions. [file EVL3-4-54-s001.pdf]

Resource-dependent evolution of female resistance responses to sexual conflict.

Wayne G. Rostant, Janet S. Mason, Jean-Charles de Coriolis and Tracey Chapman

School of Biological Sciences, University of East Anglia, Norwich Research Park, Norwich, NR4 7TJ, UK

Supplementary Information Figures

Fig S1. Frequency of courtship, mating and food occupancy during experimental evolution under male biased (MB, purple), equal sex (ES, black) and female biased (FB, orange) conditions on poor or rich resource diet regimes.

Fig S2. Response of survival to manipulation of sexual conflict and resource levels.

Fig S3. Baseline threshold survival to 32 days during no conflict assay.

Fig S4. Individual survival curves for females (red curves) and males (blue curves).

Fig S5. Ageing parameters, log initial mortality rate (α) and the rate of mortality increase (β) for MB (purple symbols), ES (black) and FB (orange) males and females.

Fig S6. Age specific fitness for fully reproductive individuals from the MB (purple), ES (Black) and FB (orange) poor and rich diet experimental evolution regimes (conflict assays).

Fig S7. Response of fitness to manipulation of sexual conflict and resource levels under conflict assay conditions.

Fig S1

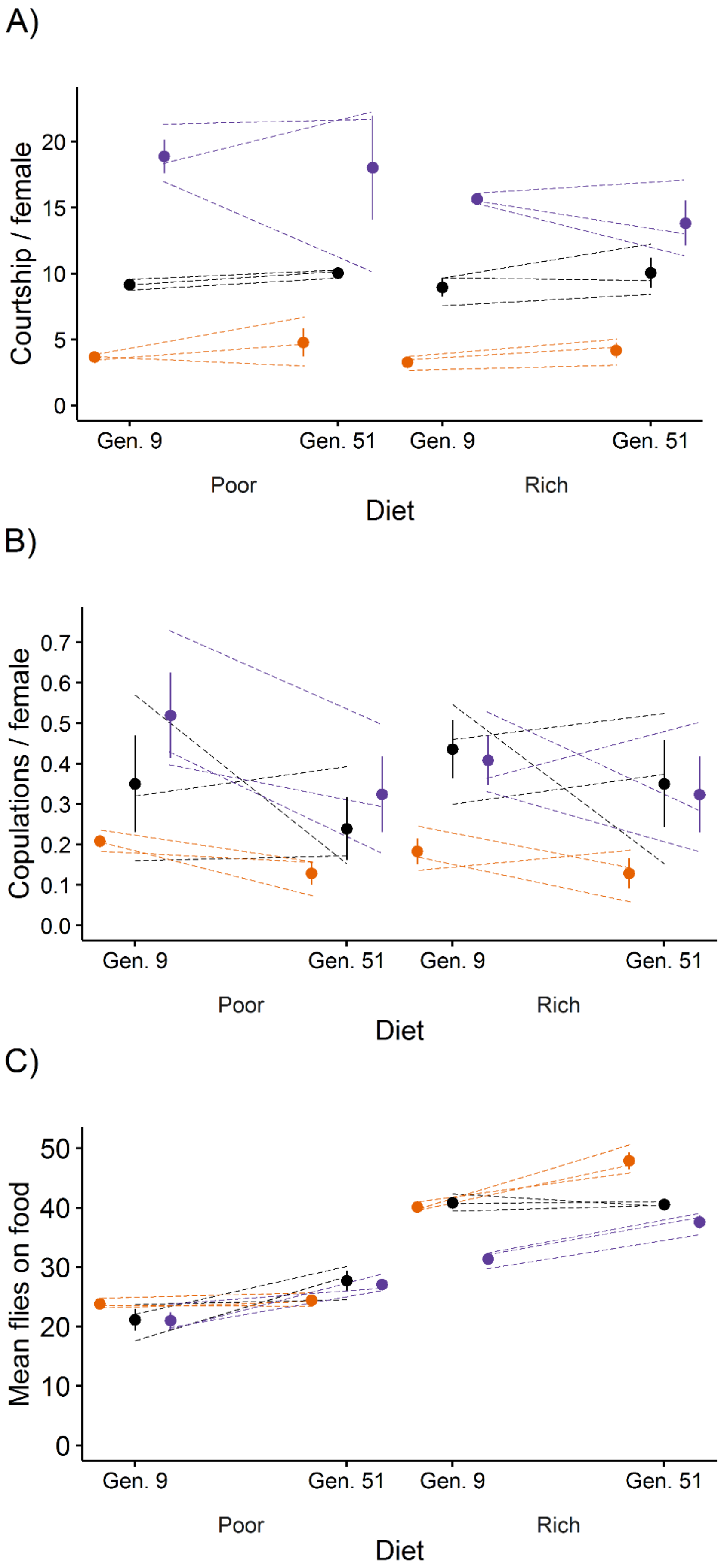
A)
Courtship / female
0
5
10
15
20
Gen. 9
Gen. 51
Gen. 9
Gen. 51
Poor
Rich
Diet
B)
Copulations / female
0.0
0.1
0.2
0.3
0.4
0.5
0.6
0.7
Gen. 9
Gen. 51
Gen. 9
Gen. 51
Poor
Rich
Diet
C)
Mean flies on food
0
10
20
30
40
50
Gen. 9
Gen. 51
Gen. 9
Gen. 51
Poor
Rich
Diet

Fig S1. Frequency of courtship, mating and food occupancy during experimental evolution under male biased (MB, purple), equal sex (ES, black) and female biased (FB, orange) conditions on poor or rich resource diet regimes. Behaviour was scored every 20 mins for 2 hours per morning over 7 days of observations during the normal 10 day adult interaction period in generations 9 and 51. Dots show averages, whiskers show 95% C.I. and dashed lines show individual replicates. **(A) Courtship events** observed per female per day, **(B) copulations** (matings) observed per female per day, and **(C) food occupancy** – average number of flies on the food per day.

Fig S2

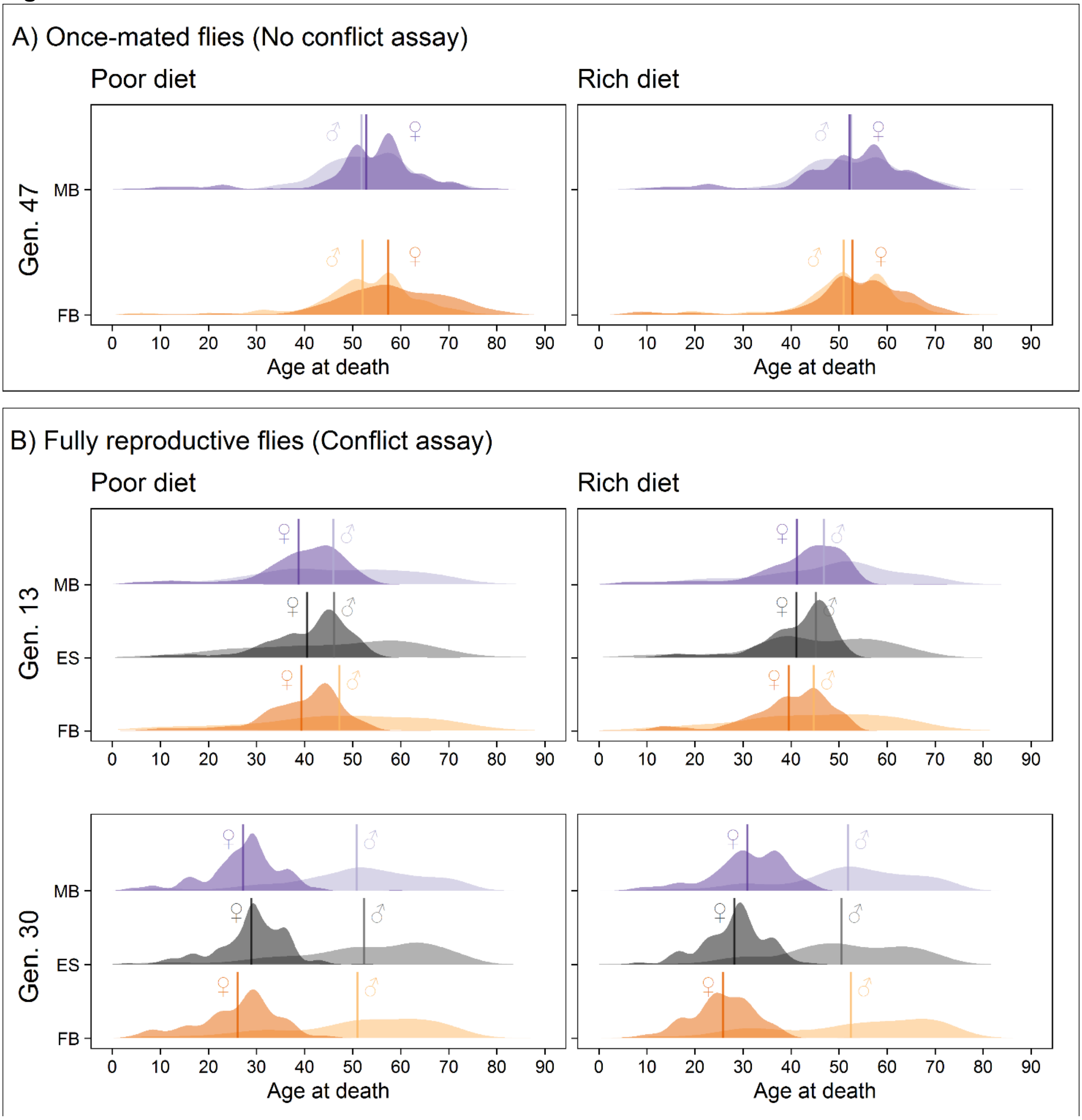

Fig S2. Response of survival to manipulation of sexual conflict and resource levels. Average lifespan for males and females is indicated by the vertical lines placed within the frequency density plots showing the distribution of lifespan for each treatment (female survival in darker colours, male survival in pale). **(A) No conflict assay**. Average base line survival of male biased (MB, purple) and female biased (FB, orange) sex ratio treatment once-mated males and females from poor and rich diet regimes following experimental evolution (gen 47). The figure highlights the higher base line survival in the FB in comparison to MB females on poor resource regimes, whereas on rich diet regimes there was no difference. Male survival did not respond to diet or sex ratio. **(B) Conflict assay**. Average survival of MB (purple), equal sex (ES, black) and FB (orange) fully reproductive males and females from the poor and rich diet regimes at generation 13 and 30 of experimental evolution. The figure highlights the increase in sex differences in lifespan as experimental evolution proceeded, and the key response of female lifespan to sexual conflict on rich, but not poor, diet regimes - with MB > ES > FB. Male lifespan again did not respond.

Fig S3

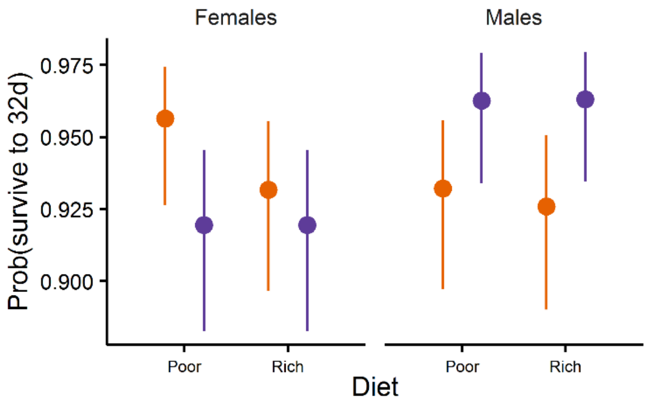

Fig S3. Baseline threshold survival to 32 days during no conflict assay. Estimated probability of survival of male biased (MB, purple) and female biased (FB, orange) sex ratio treatment once-mated females and males from poor and rich diet regimes following experimental evolution (gen 47). Points represent estimated means, whiskers 95% C.I. from GLM with binomial error family.

Fig S4

A) Once-mated, poor diet regime

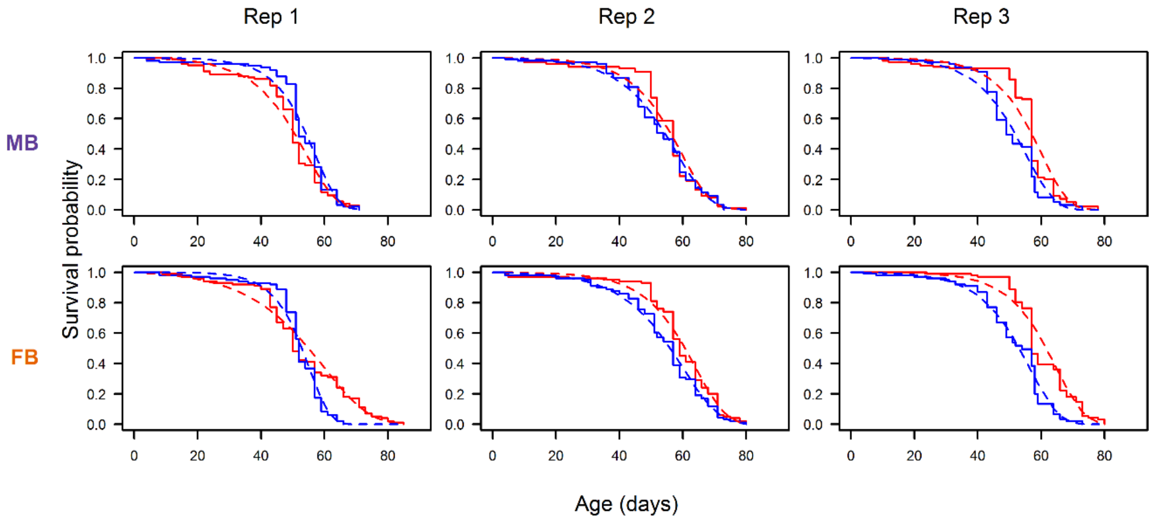

B) Once-mated, rich diet regime

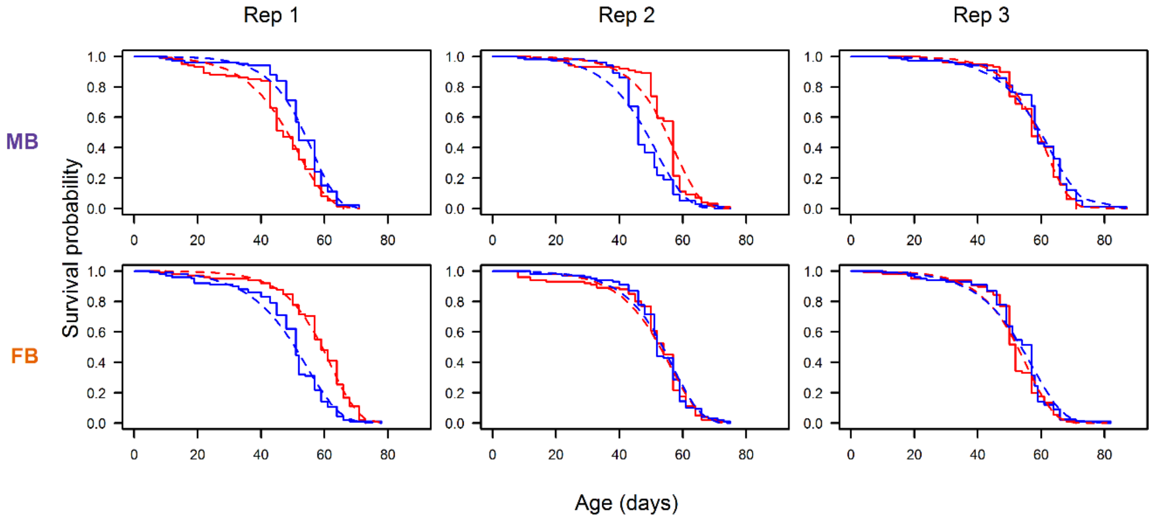

C) Fully reproductive, poor diet regime

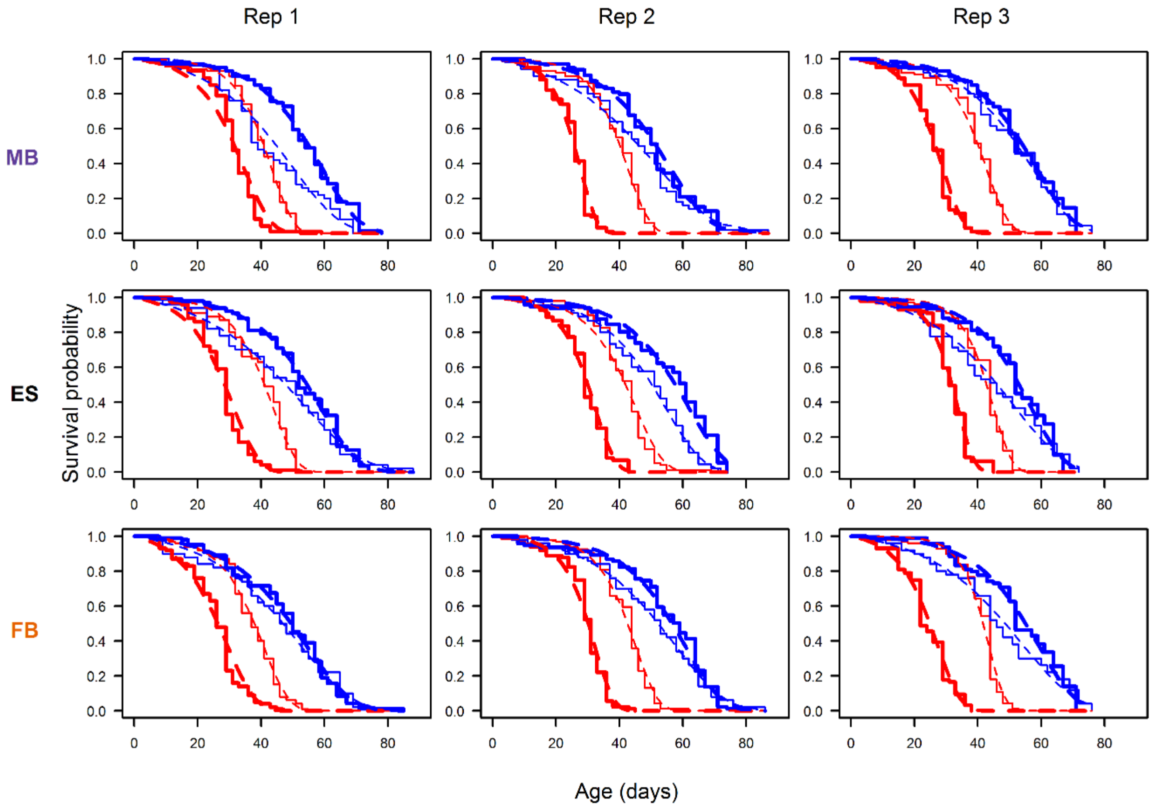

D) Fully reproductive, rich diet regime

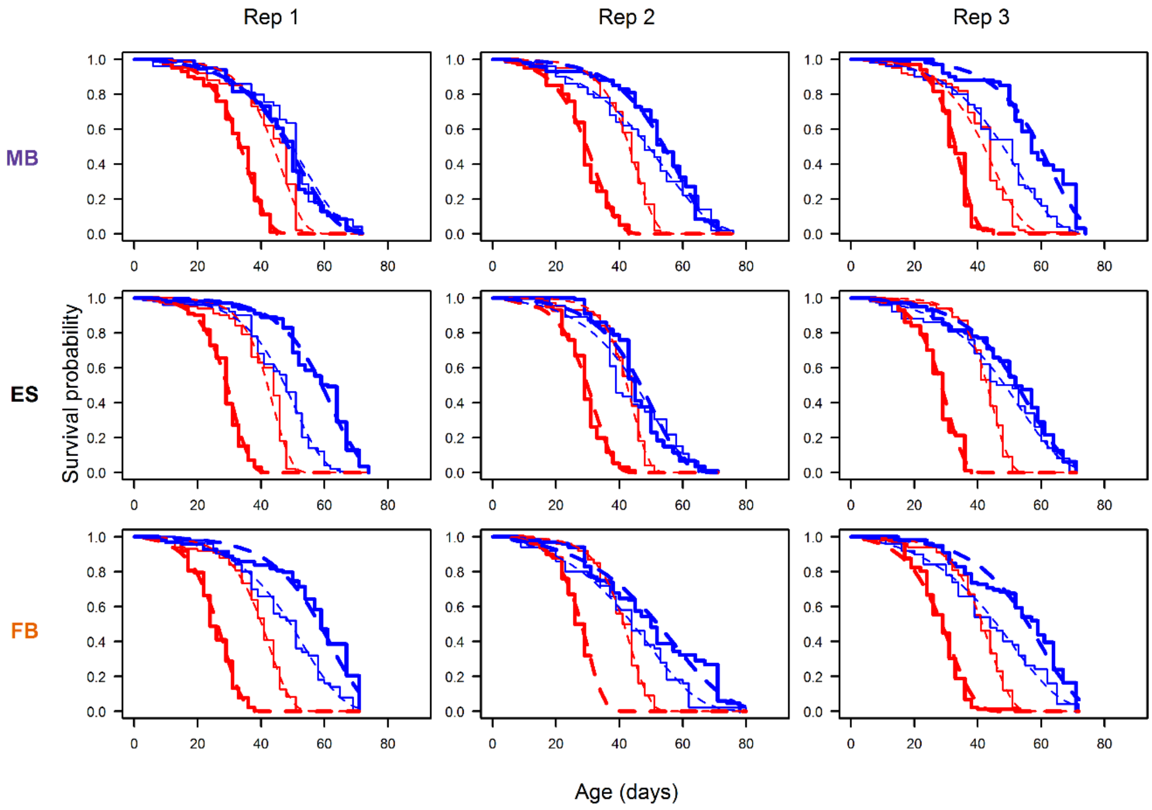

Fig S4. **Individual survival curves**: for females (red curves) and males (blue curves). Panels **(A, B)** show once-mated individuals (**no conflict assay**) (gen 47) from **(A) poor** and **(B) rich** diet regimes. Panels **(C, D)** show fully reproductive individuals (**conflict assay**) (gens 13 and 30) from **(C) poor** and **(D) rich** diet regimes. Continuous lines represent Kaplan-Meier curves. Dashed lines represent survival probability using fitted Gompertz parameters. In (C) and (D) thin lines = gen 13, bold lines = gen 30, showing the rapid evolution of sexual dimorphism for lifespan.

Fig S5

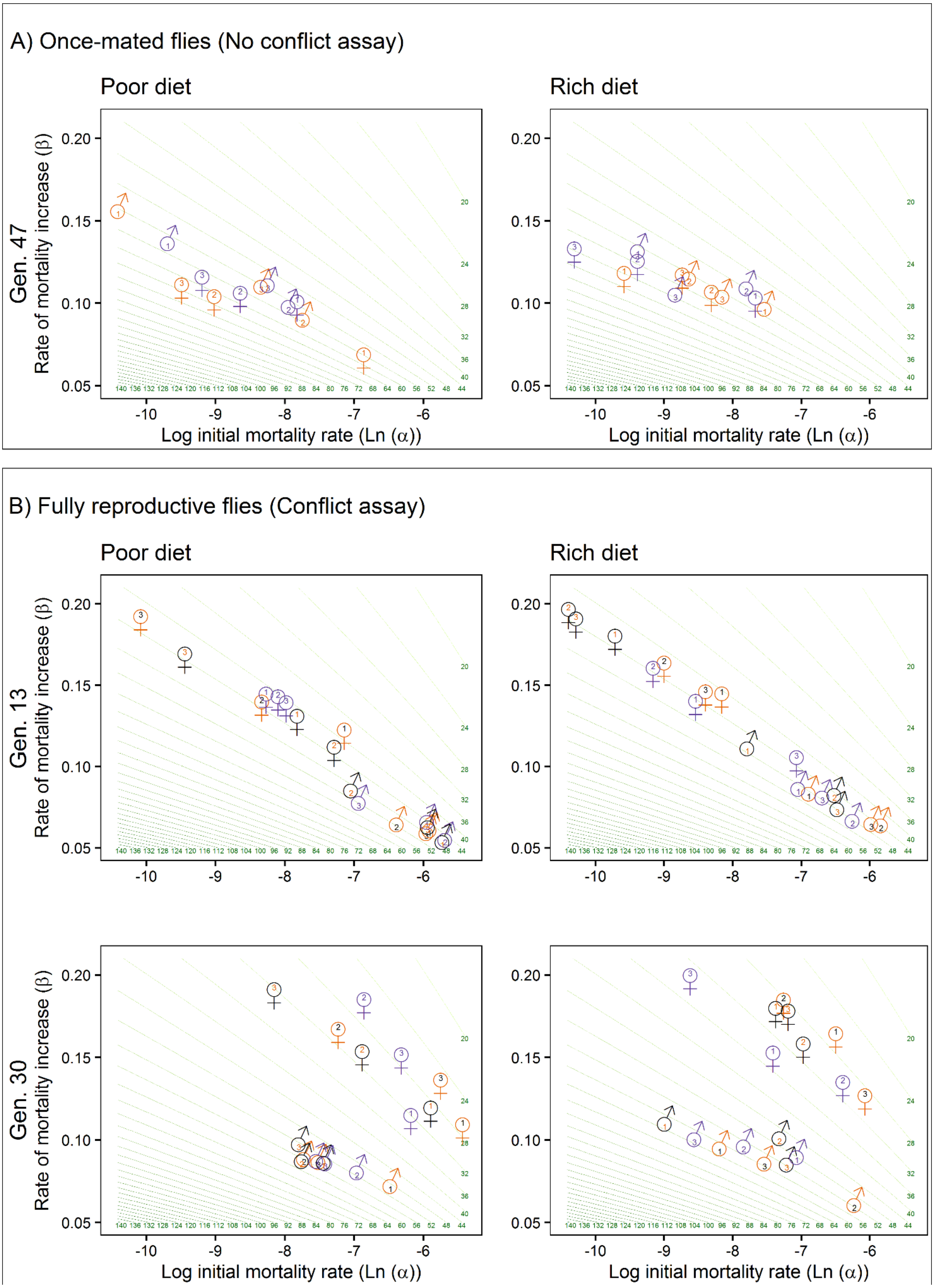

A) Once-mated flies (No conflict assay)
B) Fully reproductive flies (Conflict assay)
Poor diet
Rich diet
Gen. 47
Gen. 13
Gen. 30
Rate of mortality increase (β)
Log initial mortality rate (Ln (α))

Fig S5. Ageing parameters, log initial mortality rate (α) and the rate of mortality increase (β) for MB (purple symbols), ES (black) and FB (orange) males and females. **(A)** Base line ageing parameters for poor and rich diet regime once-mated individuals after experimental evolution (gen 47) (**no conflict assay**). **(B)** Ageing parameters in fully reproductive poor and rich diet regime individuals at generation 13 and 30 of experimental evolution (**conflict assay**), indicating the clear shift in initial mortality rate in females across generations.

Fig S6

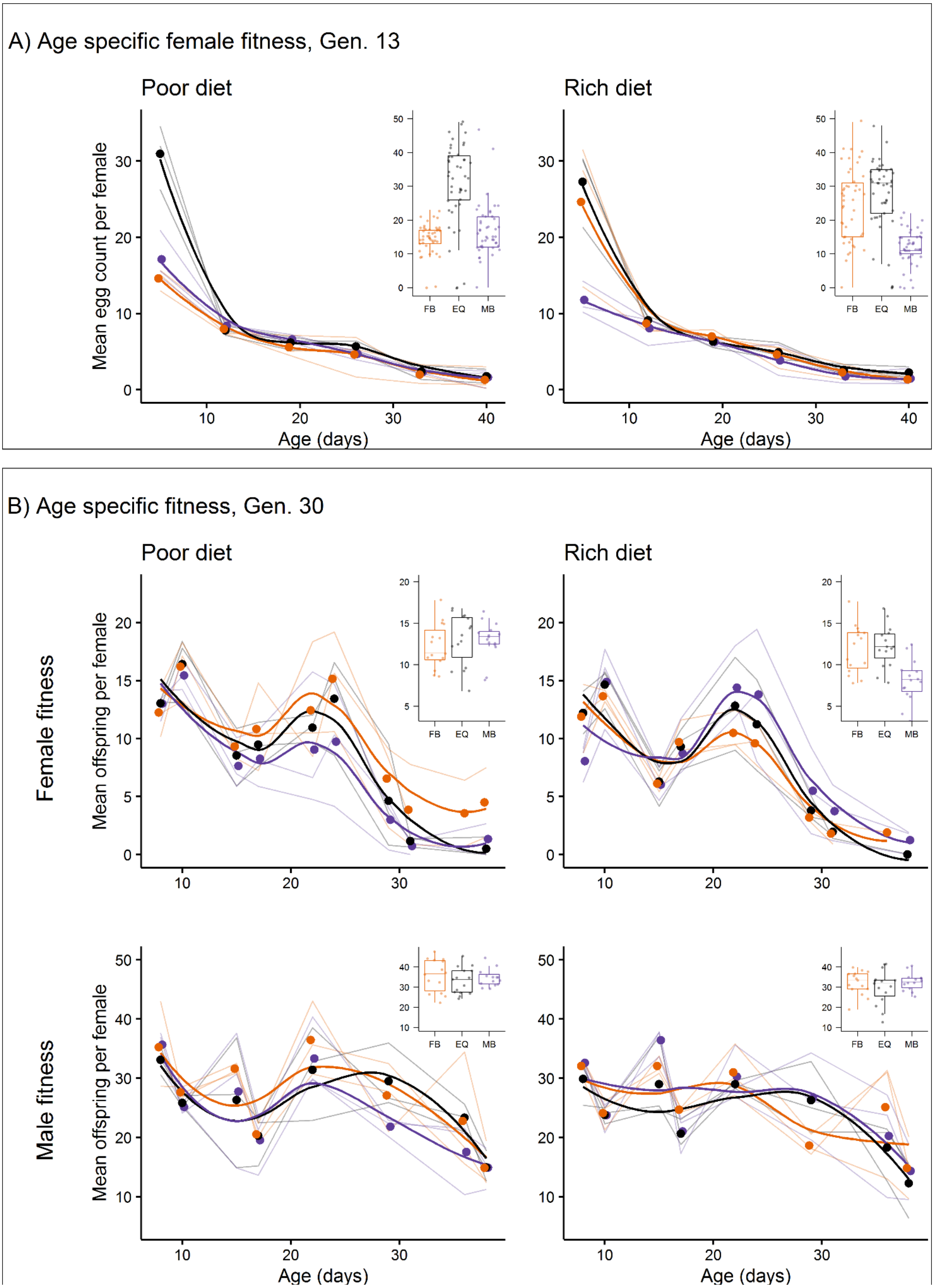
A) Age specific female fitness, Gen. 13
Poor diet
Rich diet
Mean egg count per female
Age (days)
FB
EQ
MB
B) Age specific fitness, Gen. 30
Poor diet
Rich diet
Female fitness
Male fitness
Mean offspring per female
Age (days)

Fig S6. Age specific fitness for fully reproductive individuals from the MB (purple), ES (Black) and FB (orange) poor and rich diet experimental evolution regimes (conflict assays). (A) Age specific fecundity at generation 13 for fully reproductive poor and rich diet females, given as average fecundity (±se) over the lifetime. Insets show day 5 data. (B) Age specific progeny production at generation 30 for fully reproductive females and males from the poor and rich diet regimes, given as average progeny production (±se) over the lifetime. Insets show day 8-10 data, indicating a delayed reproductive peak in MB females from the resource rich regime. In all main panels, loess was used to fit smooth curves to the age-specific fitness data. In all insets, bold horizontal lines represent median, boxes represent the interquartile range, the line whiskers represent values that are 1.5 times the interquartile range.

Fig S7

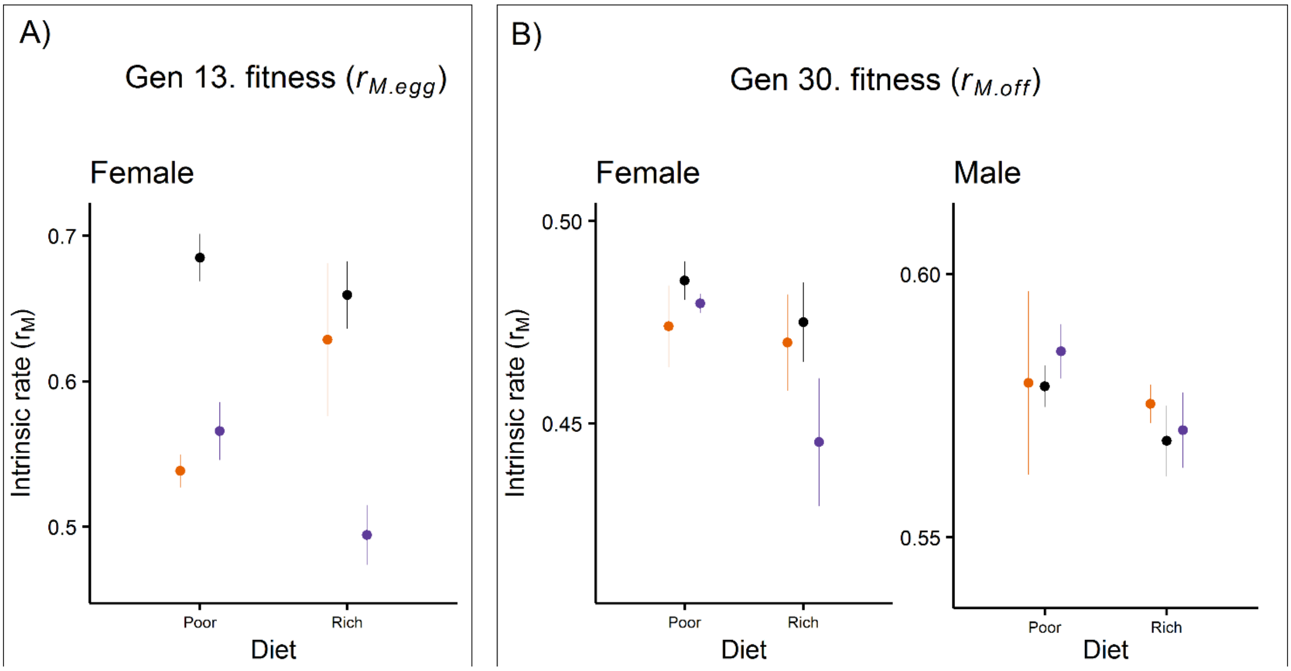

Fig S7. Response of fitness to manipulation of sexual conflict and resource levels under conflict assay conditions. (A) Female fitness early in the experimental evolution calculated as the Malthusian parameter 'r_M' (replicate means ± 95% C.I.) derived from fecundity for females from the MB (purple), ES (black) and FB (orange) poor and rich diet regimes (conflict assay conditions). **(B)** Male and female fitness following experimental evolution at generation 30, calculated as the Malthusian parameter 'r_M' (replicate means ± 95% C.I.) derived from offspring counts (conflict assay conditions). The early fitness differences disappeared by generation 30 in the poor, but not rich diet regime females, with the latter exhibiting lower fitness in the high conflict (MB) females. The lack of evolutionary response of male fitness to sexual conflict is again evident.
