# Supplementary material for: Resource‐dependent evolution of female resistance responses to sexual conflict
Source: Evol Lett. 2020 Jan 9;4(1):54–64. doi: 10.1002/evl3.153 (PMC7006461; doi:10.1002/evl3.153)
Supplement: Supplementary file 2 [file EVL3-4-54-s002.pdf]

Resource-dependent evolution of female resistance responses to sexual conflict.

Wayne G. Rostant, Janet S. Mason, Jean-Charles de Coriolis and Tracey Chapman

School of Biological Sciences, University of East Anglia, Norwich Research Park, Norwich, NR4 7TJ, UK

Supplementary Information (SI)

SI 1: Selective environment during experimental evolution – methods and results

SI 2: Detailed experimental methods

SI 3: Detailed statistical methods

SI 4: SI references

1: Measuring the selective environment during experimental evolution – methods and results

Methods

To analyse the intensity of the selective environment created by the different evolutionary regimes, courtship, mating frequency and food occupancy were measured in a series of snapshot observations in generations 9 and 51 of the experimental evolution, within the selection cages. During the morning of each of 7 days, 10 observations were made by two observers on each of the 18 cages (5 observations per observer with at least a 20min gap between each observation). During each observation every cage was viewed from three sides in turn, to minimize double counting and provide a good index of courtship activity. The number of matings and courtship events (wing display directed at a female or attempted mounting) was recorded as well as the number of flies present on the food. Any dead flies were removed and counted, to adjust sample sizes in the calculation of courtship and mating frequencies.

Results

Selective forces exerted in sexual conflict regimes maintained under rich and poor resource regimes

(i) Courtship rates per female

Stepwise simplification of the maximal linear mixed model (LMM) via parametric bootstrapping resulted in a minimum adequate model containing only the main effect of sex ratio (LRT = 36.875, N_{sim} = 10000, P < 0.0001). The number of courtships per female followed the expected pattern, with FB < ES < MB regimes (one-sided contrasts of estimated marginal means: FB < ES: t_{15} = -4.435, P_{adjust} = 0.0002; FB < MB: t_{15} = -10.045, P_{adjust} < 0.0001; ES < MB: t_{15} = -5.610, P_{adjust} < 0.0001; Fig. S1*A*). These differences were maintained throughout selection (Sex Ratio × Generation: LRT = 4.099, N_{sim} = 10000, P = 0.1873). The effects were similar on both the rich and poor yeast food (Sex Ratio × Diet: LRT = 3.8435, N_{sim} = 10000, P = 0.2818). The results show that, independent of resource levels, females in the MB regimes did indeed experience elevated courtship in comparison to the ES and FB females. The expected differences in selective forces were also maintained throughout the experimental evolution.

(ii) Mating frequency per female

Stepwise simplification as above resulted in a minimum adequate model containing the main effects of sex ratio (LRT = 14.8910, N_{sim} = 10000, p = 0.0014) and generation (LRT = 6.4596, N_{sim} = 10000, P = 0.0202). As for courtship rate, there were no significant main effects of, or interactions involving, diet. The pattern for mating frequency was similar to that of courtship rate, with females from the FB regimes receiving fewer matings than ES or MB females (one-sided contrasts of estimated marginal means: FB < ES: t_{15} = -3.274, P_{adjust} = 0.0103; FB < MB: t_{15} = -4.175, P_{adjust} =

0.0033; ES < MB: t_{15} = -0.901, P_{adjust} = 0.3819; Fig. S1*B*). In all cases, the pattern of mating frequency differences was maintained over the experimental evolution (Sex Ratio × Generation: *LRT* = 0.7001, N_{sim} = 10000, *P* = 0.7823), though mating frequency itself declined slightly overall (confirmed by one-sided contrast of estimated marginal means: Generation 51 < Generation 9: t_{17} = 2.694, P_{adjust} = 0.0205). The results show that females in the FB regimes experienced fewer matings in comparison to ES and MB females. As for courtship, resource regime had no overall effect on mating frequency (Diet effect: *LRT* = 0.0583, N_{sim} = 10000, *P* = 0.8228). Therefore, the expected differences in selective forces were maintained throughout the experimental evolution and the potential for sexual conflict was lower in FB and higher in MB lines across both rich and poor resource regimes.

(iii) Food occupancy

The minimum adequate model for food occupancy retained the sex ratio × diet × generation interaction (selenvfigfood; *LRT* = 22.107, N_{sim} = 10000, *P* = 0.0014). Removal of this interaction term revealed main effects of sex ratio (*LRT* = 11.0243, N_{sim} = 10000, *P* = 0.0118), diet (*LRT* = 46.971, N_{sim} = 10000, *P* < 0.0001) and generation (*LRT* = 16.877, N_{sim} = 10000, *P* < 0.0001). To better interrogate the interaction, the dataset was split by diet (poor versus rich) and analysed using LMMs as before. In the poor diet populations, stepwise simplification revealed a small but significant main effect of generation, with occupancy increasing slightly between generation 9 and 51 (*LRT* = 12.2739, N_{sim} = 10000, *P* = 0.0015). In contrast, stepwise simplification of the rich diet LMM revealed a significant sex ratio × generation term (*LRT* = 19.2412, N_{sim} = 10000, *P* = 0.0023) (Fig. S1*C*). For rich diet populations at generation 51 all pairwise contrasts of estimated marginal means were significant (two-sided tests: FB vs ES: $t_{11.54}$ = 5.751, P_{adjust} = 0.0002; FB vs MB: $t_{11.54}$ = 8.096, P_{adjust} < 0.0001; ES vs MB: $t_{11.54}$ = 2.345, P_{adjust} = 0.0486), but at generation 9 there was no difference in occupancy between FB and ES populations (FB vs ES: $t_{11.54}$ = -0.538, P_{adjust} = 0.6760; FB vs MB: $t_{11.54}$ = 6.876, P_{adjust} = 0.0001; ES vs MB: $t_{11.54}$ = 7.414, P_{adjust} < 0.0001). As with the poor diet LMM analysis, overall occupancy increased over time in the rich diet populations (main effect of Generation: *LRT* = 13.5825, N_{sim} = 10000, *P* = 0.0012) but there was also an overall main effect of Sex Ratio (*LRT* = 12.2739, N_{sim} = 10000, *P* = 0.0020), with food occupancy being consistently higher in FB than MB populations. In summary, resource-rich populations exhibited higher food occupancy than resource-poor populations. Food occupancy increased with time, regardless of diet but was significantly affected by sex ratio only in the rich diet populations, in which FB treatments exhibited consistently higher food occupancy than for the MB regime.

2: Detailed experimental methods

Experimental methods

The Dahomey wild-type stock used was collected in 1970 and has been maintained since then in four population cages with overlapping generations. Each Dahomey stock cage was supplied with three bottles (189 ml each) containing 70 ml of sugar-yeast (SY) food (100 g brewer's yeast powder, 50 g sucrose, 15 g agar, 30 ml nipagin (10% w/v solution), 3 ml propionic acid, 1 L water) every week. Bottles were removed after 28 days. All cultures were maintained at 25˚C in humidified rooms on a 12:12 h light: dark cycle.

Direct manipulation of sexual conflict by experimental evolution

The rationale was to create 3 replicates each of high, medium and control sexual conflict regimes by altering the adult sex ratio (male-biased, equal sex, female biased, respectively) (1). To vary adult resources we maintained adults under both rich and poor adult diets (3 regimes x 3 replicates x 2 food regimes = 18 experimental evolution treatments). The resource rich SY adult diet used represented a protein to carbohydrate level (P:C) of approximately 1:1.6 with a calorific level of 119 g P+C per litre of medium (estimated from (2)). The resource poor SY_{20} diet contained only 20% yeast, giving a P:C ratio of 1:6 and a calorific level of 64 g P+C per litre. Therefore, the poor resource level manipulation we applied in the experimental evolution halved the caloric level in addition to reducing P:C ratio by more than 3 times. Developmental diets were standardised across all regimes (all larvae were raised on the rich SY diet). Reducing caloric level (e.g. (3)) is reported to have smaller effects on adult males than females. Similar poor resource diet manipulations appear to significantly decrease female reproductive output (4) to a greater extent than is true for males (5). Thus, we grounded the experimental evolution in conditions that offered the potential for sex specific trade-offs with resistance to sexual conflict to operate in females under poor, but not rich resource regimes.

The experimental evolution was initiated by collecting eggs from four replicate Dahomey cages laid onto Petri dishes filled with a grape juice medium (50 g agar, 600 ml red grape juice, 42.5 ml Nipagin (10% w/v solution), 1.1 L water) containing yeast paste. First instar larvae were placed in batches of 100 into glass vials (75 mm height x 25 mm diameter) each containing 7 ml of SY food and live yeast. After all flies had eclosed, the adults were mixed, sexed under CO_2 anaesthesia, and randomly allocated to one of the 18 treatments. The diet regimes consisted of rich SY versus poor SY food (SY_{20}). For each diet, three replicate lines each of male biased (MB, 70 males and 30 females), equal sex (ES, 50 males and 50 females), and female biased (FB, 25 males and 75 females) sex ratio treatments were created. Each line was maintained in a 1L plastic tub with a gauze-covered top. Flies had access to water via two water-filled vials with cotton wool wicks and were fed with two SY or SY_{20} vials every 2-3 days. Nine days after the cages were set

up (with adults aged 10 – 11 days post eclosion) eggs were collected from each cage on yeasted grape plates. Three hundred first instar larvae of each line were picked and raised at standard density (100 larvae per vial) on SY food. Standard density culturing for all lines minimized competition at the larval stage and differential developmental conditions. To minimize selection on development time, all adults were allowed to eclose over two days before being allocated to the same diet x sex-ratio treatment and replicate number as their parents. All subsequent generations were maintained using the same protocol.

Effect of high and low sexual conflict on intrinsic mortality, ageing and lifespan in once-mated males and females ('no conflict' assay)

To test the effects of elevated sexual conflict we measured the baseline ageing and rate of ageing of once-mated males and females from the high (MB) and low (FB) regimes from both resource levels following experimental evolution, at generation 47 (2 sex ratio treatments x 2 nutritional regimes x 2 sexes x 3 replicates). Individuals were mated once at the start of their lives, hence the assay itself did not incorporate the effects of sexual conflict (hence, 'no conflict' assay). Standard (rich) SY medium was used throughout. One day after egg collection for selection line maintenance (i.e. with adults 11 – 12 days post eclosion) eggs were collected from each selection cage on yeasted grape plates. 300 first instar larvae of each line were picked and raised at standard density (100 larvae per vial) on SY medium. Adults were collected upon eclosion and housed in single sex vials of 10. 50 individuals from each focal sex from each treatment were then housed with 50 wildtype individuals of the opposite sex in a single culture bottle (2 bottles per sex per treatment) for 24h, to ensure they were all 'once-mated'. Individuals were then separated and divided into single-sex groups of 5 per vial (20 vials per focal sex per treatment giving a sample size of n = 100 per focal sex per treatment overall). The groups of 5 individuals per vial were transferred onto new food every 2-3 days. Individuals were shuffled between vials once per week, maintaining the same density of 5 flies per vial. Mortalities were recorded every day until all flies were dead.

Effect of sexual conflict on intrinsic mortality, ageing and lifespan in fully reproductive males and females at gen 13 and after 30 gens of experimental evolution ('conflict assay').

To test the effects of elevated sexual conflict we measured the baseline ageing and rate of ageing of fully reproductive males and females from the high (MB) and low (FB) regimes from both resource regimes at generation 13 and 30 of experimental evolution (2 sex ratio treatments x 2 nutritional regimes x 2 sexes x 3 replicates x 2 time points). Individuals were maintained continually with standard wild individuals of the other throughout their lives, hence the assay incorporated the effects of sexual conflict (hence, 'conflict assay').

At generations 13 and 30, experimental flies were generated as described above, i.e. newly eclosed adults from each of the regimes were placed within small laying cages with a grape juice

agar plate (at age 3 to 4 days old). The larvae derived from these laying plates were then placed into standard SY vials at a density of 100. Adults emerging from these cultures were collected as virgins and aged for 1 day in single sex groups of 10. Simultaneously, cultures of standard density wild type flies were set up, to provide the non-focal individuals. All individuals were separated upon eclosion and divided into their experimental treatments of 5 focal individuals + 5 wild type per vial (20 vials per focal sex per treatment giving a sample size of n = 100 per focal sex per treatment overall). The groups of 5 + 5 individuals per vial were transferred onto new food every 2-3 days. Individuals were shuffled between vials once per week, maintaining the same density of 5 focal + 5 wild type flies per vial. Wild type individuals were replaced from fresh cultures every week. Mortalities were recorded every day until all flies were dead.

We also analysed mating behaviour in these assays, at gens 13 and 30. Courtship and mating frequencies were measured in a series of weekly snapshot observations. On one morning every week, 6 observations were made on each vial (with at least a 20min gap between each observation). During each observation every female fly was viewed in turn, to minimize double counting and provide a good index of courtship activity. The number of matings and courtship events (wing display directed at a female or attempted mounting) was recorded. Any dead flies were noted, and only vials with a full complement (5 females and 5 males) alive were used for subsequent analysis.

3. Detailed statistical analyses

Lifespan

Linear mixed (LMM) models were used for lifespan analyses as a well-established and flexible approach to dealing with non-independent data (random effects) and this method also allowed us to avoid the challenges due to the occurrence of non-proportional hazard ratios. Though these models do not incorporate censors, these were very rare and occurred randomly across treatments. Their exclusion did not bias the analyses. Our subsequent age parameter analyses also allowed us to look at the patterns of demographic ageing. All survival analyses were conducted using R (v. 3.3.2 (6)). In each case, stepwise simplification of the maximal models was conducted.

Model specification and suitability:

(i) Once-mated flies (no conflict assay, gen 47). Due to the very low early-life mortality in all treatments during the once-mated experiment the residuals of the initial full linear mixed effects model revealed heavy left-skew that was not amenable to suitable transformation. Instead, we analysed the data in two stages by using an age threshold which balanced the desire to eliminate skew while maximizing the data subjected to the LMM approach. In the first stage we examined the proportion of flies surviving to age 32 days. Initially we fitted this using a binomial GLMM with survival to age 32 as a binary response and population as a random effect. However, the random effect of population was estimated as zero in the maximal model, suggesting overfitting. Therefore, we proceeded with a simpler Generalized Linear Model (GLM) without any random effect. In the second stage we analysed the lifespan of all flies that survived to at least age 32 days i.e. excluding 145 (6%) uncensored individuals that died before age 32. Examination of the residuals for the maximal LMM model in this stage showed homoscedasticity and normal distribution of residuals and dAIC scores showed a superior fit of LMM to both Poisson and negative binomial error GLMM specification.

(ii) Fully reproductive flies (conflict assay, gens 13 and 30). Examination of residuals in the initial full linear mixed effects model revealed considerable left-skew due to relatively low early-life mortality and heteroscedasticity due to characteristically different variances in age-at-death for males and females (Focal Sex) and between time points (Generation) sampled (see ‘Ageing Parameters’ below: illustrated by differences in ageing parameters). Thus, prior to further analysis, age-at-death was square-transformed to correct the skew and variance was explicitly modelled as a function of Focal Sex and Generation. Model fit was satisfactorily improved by these measures and dAIC scores showed a superior fit of LMM to both Poisson and negative binomial error GLMM specification.

Testing of fixed effects:

Fixed effects were tested by stepwise model simplification from the maximal model (which included relevant main factors and higher order terms up to three-way interactions) to a minimum adequate model using parametric bootstrap to compare nested models (i.e. models with and without the effect in question). This was implemented using the PBmodcomp() function from the 'pbkrtest' package (7). Under the fitted hypothesis (i.e. under the fitted smaller model, without the effect in question) 10000 samples of the likelihood ratio test statistic (LRT) were generated by fitting both large and smaller model. Then p-values are calculated as the fraction of simulated LRT-values that are larger or equal to the observed LRT value.

Mating frequency of fully reproductive individuals (conflict assays)

We tested whether treatment had any effect on the age specific sum of mating and courtship events experienced by females within different treatments. Because individual females were not followed within vials and due to weekly shuffling among vials within each population, only population-level measures were possible. Cumulative courtship and copulation indices were derived as follows. For each week of observations, the average per vial count for each behaviour was calculated for each population. Thus for each of the 18 populations a cumulative courtship index and cumulative copulation index was calculated at each sampling age. Only time-points with at least one full complement vial from each population were used in statistical analysis. 5 weekly measures were available from generation 30 and 6 from generation 13. To make analyses more tractable, and due to differences in the functional form of cumulative mating, separate LMMs were built for each diet x generation subset of the data. In all four models, population was included as a random intercept effect.

Ageing parameters (α and β)

Model specification and suitability:

Age-specific mortality data for each focal sex within each population at each generation (13, 30 and 47) were fitted with a series parametric two parameter models. Comparisons using AIC showed that overall, the Gompertz mortality function was the best fit to the datasets for the data. Under this model mortality is fitted as:

$$\mu(x) = \alpha e^{\beta x}$$

where x is age (days), $\mu(x)$ is age-specific mortality, α is background mortality and β is rate of mortality increase per day. Overlay of the parametric survival curves for each population × sex × generation are shown on the empirical Kaplan-Meier plots (Fig. S4).

The ageing parameters were negatively correlated (Fig. S5), hence we modelled their response to treatments using a multivariate framework as follows. Due to violation of traditional MANOVA assumptions, we used a non-parametric (permutational) MANOVA (8), as implemented by the adonis2() function in the 'vegan' package (9). Order-of-magnitude differences in mean and

variance between α and β parameters were minimized by scaling each response prior to analysis and Euclidean pairwise distances were calculated prior to permutation significance tests with pseudo-*F* ratios. The hierarchical structure of the data necessitated a restricted permutation scheme whereby permutation of treatment assignments to generate a null distribution always kept flies from the same population x generation together.

Testing of fixed effects was conducted by model simplification proceeded by stepwise elimination of non-significant factors, where significance was determined by marginal effects. Multivariate dispersion (akin to multivariate variance) was compared for all factors and interactions using the betadisper() and permutest() functions in the 'vegan' package and the same null distribution as the permutational MANOVA. This was used to inform whether treatment differences were solely due to location and/or spread of the data.

Ageing rate index (ω_G)

Model specification and suitability: An ageing rate index ω_G (10) was derived from the fitted Gompertz parameters (per sex per population per time point) as follows:

$$\omega_G = (\alpha\beta)^{-1/2}$$

By incorporating both Gompertz parameters, this index indicates the overall magnitude of the mortality rate at a particular age and retains the correct units (day^{-1}), thereby being a useful index of ageing. For both datasets (once-mated and fully reproductive) this index was modelled as the response in LMMs where maximal models included all relevant fixed factors and higher order interactions of these factors up to and including any 3-way interactions. In the once-mated (no conflict assay) dataset (gen 47), population was included as the random intercept term, while for the fully reproductive (conflict assay) dataset (gens 13 and 30) we also modelled repeated measures per population (two generations) as a random slope, where random slope and intercept were correlated. Testing of fixed effects was achieved by stepwise model simplification from the maximal model to a minimum adequate model as described in 'Lifespan' section above.

Fitness data

A modification of growth curve analysis (11) was used to analyse the fitness data (age-specific egg or adult offspring counts per female) as follows. Full mixed effects models (LMMs or GLMMs, with most parsimonious error family determined via AIC) specified the overall time course of offspring production with a fourth-order (quartic) orthogonal polynomial and fixed effects of sex ratio treatment on all time terms. This functional form was suitable for the non-linear aspect of offspring production over the lifespan. To render the analysis tractable and to allow model convergence, full datasets were split by diet and, where appropriate, focal sex. The full models also included population random effects on as many time terms as possible, except where limited by model convergence constraints. Model simplification proceeded via stepwise simplification first of the

random effects and then fixed effects. Random terms and fixed effects were either tested using analysis of deviance (for egg count GLMMs at generation 13) or parametric bootstrapping (for adult offspring count LMMs at generation 30).

Female and male fitness indices were calculated as the intrinsic rate of population growth (the Malthusian parameter, Euler's r), using the Euler equation (12, 13), separately for each treatment line. The Euler equation calculates an index of fitness from age-specific survivorship and age-specific reproduction values and is weighted towards early-life reproduction and is directly related to the lambda fitness metric (14, 15). Age-specific egg counts (per female per 24h) were used to calculate fitness indices for generation 13 females and age-specific offspring counts (per 48h) for generation 30 males and females.

4. SI References

1. Wigby S, Chapman T (2004) Female resistance to male harm evolves in response to manipulation of sexual conflict. *Evolution* 58:1028-1037.
2. Lee WC. Micchelli CA (2013) Development and characterization of a chemically defined food for *Drosophila*. *PLoS One* 8:e67308.
3. Magwere T, Chapman T, Partridge L (2004) Sex differences in the effect of dietary restriction on lifespan and mortality rates in female and male *Drosophila melanogaster*. *J Gerontol: Biol Sci* 59A:3-9.
4. Chapman T, Partridge L (1996) Female fitness in *Drosophila melanogaster*: an interaction between the effect of nutrition and of encounter rate with males. *Proc Roy Soc B* 263:755-759.
5. Adler MI, Cassidy EJ, Fricke C, & Bonduriansky R (2013) The lifespan-reproduction trade-off under dietary restriction is sex-specific and context-dependent. *Exp Gerontol* 48:539-548.
6. Team RDC (2016) R: A language and environment for statistical computing. R Foundation for Statistical Computing, Vienna, Austria.
7. Halekoh U, Højsgaard S (2014) A Kenward-Roger approximation and parametric bootstrap methods for tests in linear mixed models - the R package pbkrtest. *J Stat Software* 59:1-30.
8. McArdle BH, Anderson MJ (2001) Fitting multivariate models to community data: a comment on distance-based redundancy analysis. *Ecology* 82:290-297.
9. Oksanen J*, et al.* (2018) Vegan: community ecology package. R package version 2.4-6.
10. Ricklefs RE & Scheuerlein A (2002) Biological implications of the Weibull and Gompertz models of aging. *J Gerontol A: Biol Sci Med Sci* 57:B69-B76.
11. Mirman D (2014) Growth curve analysis and visualization using R. *Chapman and Hall / CRC.*
12. Gotelli NJ (2001) A Primer of Ecology. *Sinauer, Sunderland, MA*.
13. Wigby S, Chapman T (2005) Sex peptide causes mating costs in female *Drosophila melanogaster. Curr Biol* 15:316-321.
14. McGraw JB, Caswell H (1996) Estimation of individual fitness from life-history data. *Am Nat* 147:47-64.
15. Brommer JE, Merila J, Kokko H (2002) Reproductive timing and individual fitness. *Ecol Lett* 5:802-810.
